# Analyzing the Communication Interchange of Individuals With Disabilities Utilizing Facebook, Discussion Forums, and Chat Rooms: Qualitative Content Analysis of Online Disabilities Support Groups

**DOI:** 10.2196/12667

**Published:** 2019-09-30

**Authors:** Nichole E Stetten, Kelsea LeBeau, Maria A Aguirre, Alexis B Vogt, Jazmine R Quintana, Alexis R Jennings, Mark Hart

**Affiliations:** 1 College of Public Health and Health Professions University of Florida Gainesville, FL United States; 2 Bouvé College of Health Sciences Northeastern University Boston, MA United States

**Keywords:** persons with disabilities, social media, social support, online social networking, internet, psychosocial support systems, qualitative research

## Abstract

**Background:**

Approximately 1 in 5 adults in the United States are currently living with a form of disability. Although the Americans with Disabilities Act has published guidelines to help make developing technology and social networking sites (SNS) more accessible and user-friendly to people with a range of disabilities, persons with disabilities, on average, have less access to the internet than the general population. The quality, content, and medium vary from site to site and have been greatly understudied. Due to this, it is still unclear how persons with disabilities utilize various platforms of online communication for support.

**Objective:**

The objective of this study was to qualitatively explore and compare the interactions and connections among online support groups across Facebook, discussion forums, and chat rooms to better understand how persons with disabilities were utilizing different SNS to facilitate communication interchange, disseminate information, and foster community support.

**Methods:**

Facebook groups, discussion forums, and chat rooms were chosen based on predetermined inclusion criteria. Data collected included content posted on Facebook groups, forums, and chat rooms as well as the interactions among group members. Data were analyzed qualitatively using the constant comparative method.

**Results:**

A total of 133 Facebook posts, 116 forum posts, and 60 hours of chat room discussions were collected and analyzed. In addition, 4 themes were identified for Facebook posts, 3 for discussion forums, and 3 for chat rooms. Persons with disabilities utilized discussion forums and chat rooms in similar ways, but their interactions on Facebook differed in comparison. They seem to interact on a platform based on the specific functions it offers.

**Conclusions:**

Interactions on each of the platforms displayed elements of the 4 types of social support, indicating the ability for social support to be facilitated among SNS; however, the type of social support varied by platform. Findings demonstrate that online support platforms serve specific purposes that may not be interchangeable. Through participation on different platforms, persons with disabilities are able to provide and receive social support in various ways, without the barriers and constraints often experienced by this population.

## Introduction

### Overview

Currently within the United States, more than 20% of adults are living with some form of disability [1]. As defined by the Centers for Disease Control and Prevention, a disability is “any condition of the body or mind (impairment) that makes it more difficult for the person with the condition to do certain activities (activity limitation) and interact with the world around them (participation restrictions)” [1]. Although living with a disability can impact participation in many parts of your life, it does not prevent most persons with disabilities from participating in information sharing, participating in community engagement, and providing support on social networking sites (SNS). In fact, research suggests the use of SNS is generally high among persons with disabilities [2], which includes SNS such as online discussion forums, chat rooms, and Facebook.

With developing technology and SNS, the ease of communication has drastically increased over the last few years, and the internet has become an increasingly common platform for the formation of electronic peer-to-peer, or online, communities [3,4]. Online communities are social networks formed or facilitated by means of a technical platform (eg, Facebook, discussion forums, and chat rooms) through which groups of people with similar interests can establish social relationships and connect and interact with one another [3,5]. Through the development and utilization of online communities, we have seen a shift in the way social support is sought, organized, and communicated, resulting in online communities that function similarly to physical, or in-person, communities [6,7].

### Social Support and Online Communities

Social support is a theoretically complex and multidimensional construct that is often defined as the “aid and assistance exchanged through social relationships and interpersonal transactions” [8-10]. Although in-person social support has been of interest to researchers for several decades, a shift toward investigating and understanding social support in an online context has begun to occur. Online social support has been defined as the internet-facilitated receipt of both tangible and intangible assistance from people in one’s social network [6,11]. Numerous studies have found that online communities provide a platform for social support to be communicated [4,5,12-14] and that similar types of social support found in offline settings also exist in online contexts [4,6,13,14].

Social support, whether in-person or online, can generally be divided into structural and functional aspects [6,15,16]. Structural aspects include the extent to which individuals are situated within or integrated into social networks. This can be the size and structure of a social network, such as density and composition, social integration, or embeddedness [15,17-19]. Functional aspects include the psychological and material resources available from an individual’s interpersonal relationships. Functional aspects refer to the types of social support, such as esteem and emotional, informational, instrumental support, and belonging [4,6,9-11,15,17-19]. Definitions for each type of social support can be found in Table 1. The provision of social support is considered one of the important functions of social relationships [9,10], which can be measured by structural support, functional support, or a combination of both.

**Table 1:** Social support types and their accompanying definitions.

Type of social support	Definition
Esteem and emotional support	Communications from others that convey being held in high esteem, offering help with one’s emotional state, or expressing acceptance, caring, liking, respect, concern, empathy, or sympathy [6,9,10,19,20]
Informational support	Offering help in the form of advice, constructive feedback or affirmation, new information or perspectives, or references to new resources [6,9,10,19,20]
Instrumental support	Provision of tangible aid and services, such as offering financial aid, providing material resources, or taking on a responsibility [6,9,10,19]
Belonging support	Conveys a sense of social belonging and having others to engage with in shared social activities [6,9]

The benefits of social support have been repeatedly affirmed in the literature [13]. Social support has been associated with predicting and promoting good physical and mental health, reducing and preventing illness, moderating life stress, and improving quality of life [8,15,17,18,21]. In general, however, persons with disabilities are more likely to have limited access to social support and its benefits. They may experience a lack of access to social support because of misconceptions about disabilities, stigma surrounding disability, feelings of embarrassment or social isolation, and physical barriers [16,22].

The internet could circumvent these barriers by providing a useful alternative for persons with disabilities to access social support and interact in a way that may not be possible offline [13,14,22]. Due to the availability and proliferation of online communities, a new outlet for social support can be accessed. Persons with disabilities can utilize the internet’s extensive communication capabilities to access information and develop online support groups with other persons with disabilities through online platforms [13,22]. The formation of these groups may not only offer support and community-building but also link users to an increasing amount of resources, knowledge, services, shared experiences, and social exchanges [3-5]. In addition, computer-mediated environments afford them the ability to break down physical and geographic barriers to participation, including the constraints of time and distance, which might otherwise exist [16,23].

### Objective

Although it is known that persons with disabilities utilize SNS [2], it is still unclear how they utilize the various forms of online communication for support. There is a lack of research qualitatively assessing the interactions occurring among individuals within these online communities as well as the variation of quality and content from site to site [13]. With the rise in popularity of online support groups and the ever-changing nature of the internet, there is a need to explore the experiences of persons with disabilities in various supportive communication settings. The objective of this study was to explore and compare the interactions and connections among online support groups to understand how persons with disabilities are utilizing different SNS to gather and disseminate information and foster community support. Specifically, 3 SNS were selected for comparison: (1) Facebook groups, (2) online discussion forums, and (3) online chat rooms. These platforms were selected because of their popularity as platforms for the formation of online support group communities [3,5,14,20]. All 3 platforms offer users distinct environments for various types of social support to be exchanged. In addition, social support has been shown to exist among each of these online platforms to some degree [4,5,18,20].

## Methods

### Description of Social Networking Sites

Due to its popularity and accessibility, Facebook has become a common platform for the organization of online support groups [24]. Facebook offers both synchronous and asynchronous features to its users, such as the ability to react to, comment on, or share a post. Compared with online discussion forums and online chat rooms, Facebook is a less anonymous platform. Although the use of Facebook as a means for Web-based interaction has increased in popularity over the past decade, online discussion forums are still regularly used by approximately 20% of online users in the United States [25]. Discussion forums are an asynchronous communication platform whereby one person writes a post which is then answered by other members, thus creating a thread of posts related to one subject [14]. Discussion forums are a common platform for online support groups. They have been shown to be a useful source of support [14,25] and to be helpful to users because they can provide connections to others with similar experiences [26]. Online chat rooms that cater specifically to persons with disabilities also exist [22]. Chat rooms are a synchronous form of communication, that is, communication occurs in real time, and simultaneously, between users. Both discussion forums and chat rooms afford their users anonymity [25].

### Data Collection

The study was approved by the Institutional Review Board of the University of Florida. During the approval process, the information being obtained for this study was deemed *public record* and, therefore, exempt from informed consent. All data were deidentified before being analyzed by the researchers.

#### Facebook Groups

General disability support groups were targeted for the study to increase the generalizability of the information posted. These were groups that did not identify themselves for a specific disability (ie, epilepsy and multiple sclerosis). The term *disability support group* was searched using the Facebook search bar function. The Facebook groups had to have at least 6 months’ worth of data to collect to be included in this study.

In total, 3 disability support groups on Facebook were initially identified according to group type (general disability support group) and group size. Names of the specific groups have not been included to protect all individuals’ identities. Upon review of the identified support groups, only 1 group fully met our inclusion criteria of having at least 6 months’ worth of data to collect. Therefore, the other 2 groups were excluded from this study. The included Facebook group had 11,765 followers at the time of the study and was an online support community for anyone who had a disability or supported someone with a disability. Screenshots of posts, comments to posts, and reactions on posts were captured to assist with analyses. After 6 months’ worth of data were collected retrospectively, there were a total of 133 Facebook posts, all of which were analyzed. All postings were deidentified to protect participants’ identities.

#### Discussion Forums

The discussion forums were selected through the Google search engine using the keywords *disability* and *disability support group*. The discussion forums had to be publicly available and have active discussion to be included in the study. Out of the 4 discussion forums initially identified, only 2 met the inclusion criteria. There was no set time period for data collection from the forums. Discussions from the designated forums were chosen based on high activity levels. Discussions from the forums were copied and pasted verbatim for analysis. A total of 116 discussion forum posts were collected and analyzed. All postings were deidentified to protect participants’ identities.

#### Chat Rooms

Similar to the discussion forums’ selection, online chat rooms were chosen through the Google search engine using the keywords *disability* and *disability support group*. Chat rooms had to be publicly available and have active participation to be included in the study. A total of 2 chat rooms were selected, both meeting the inclusion criteria. It was predetermined by the researchers that a minimum of 60 hours of live session chat room data should be collected. Chat room data were collected during live sessions at various times on weekdays (Monday through Friday) and weekends (Saturday and Sunday) to ensure that all forms of conversations and all active participants were captured in the data collection process. To capture an accurate representation of communications occurring among chat room users, the researchers collected live session chat room data at different times of the day: 20 hours of data were collected in the morning (8:00 am-11:00 am) and early afternoon (11:00 am-1:00 pm); 20 hours of data were collected in the midday (1:00 pm-4:00 pm) and evening (4:00 pm-9:00 pm); and 20 hours of data were collected at night (9:00 pm-12:00 am). Each of these time points were collected on each day of the week. Discussions from the online chat rooms were copied and pasted verbatim for analysis. All postings were deidentified to protect participants’ identities.

### Data Analysis

The constant comparative method was used to analyze the content from the Facebook support group, discussion forums, and chat rooms to reduce the data into manageable units and coded information [27-29]. To begin this process, trained researchers independently open-coded the Facebook posts (AJ and JQ) and the discussion forum and chat room posts (AV and MA). Open coding has been defined as “the process of breaking down, examining, comparing, conceptualizing, and categorizing data” [27-29]. Upon completion of open coding, major themes and subthemes were carefully and purposefully developed from these codes for the Facebook, discussion forum, and chat room posts. Coding was continued until saturation of the data was met and no new themes emerged [27-29]. To accurately represent the discussions persons with disabilities had on each platform, the information users posted was not fact-checked by the researchers. This was decided as acceptable by the researchers because of the focus of this study being on the content of what was being posted and shared and the ways persons with disabilities utilized platforms, not on the accuracy of what was being posted.

## Results

### Facebook Support Group

All Facebook support group posts were deidentified, and no user names were included. Instead, quotes are presented as blockquotes with the participant identifier as [User post] after the quoted text to indicate an original comment. Quotes taken from the Facebook support group underwent minor modifications, such as corrections to spelling or grammatical errors and removal of explicit language. The researchers decided to modify posts in this way to enhance readability of the posts, alleviate any confusion to the reader, and protect the privacy of all users. Quotes were only modified as long as the context of the post did not change.

Among the 133 posts analyzed, the constant comparative method revealed 4 major themes, as displayed in Textbox 1. The 4 themes that emerged through analysis of the Facebook support group included mutual and shared experiences, societal concerns, awareness, and health care policy (Textbox 1).

**Disability support group themes from Facebook, discussion forums, and chat rooms.**

Themes:

Online platform: Facebook

- Mutual and shared experiences
- Societal concerns
- Awareness
- Health care policy

Online platform: discussion forums

- Emotional outlet and support
- Health
- Quality of life

Online platform: chat rooms

- Emotional outlet and support
- Health
- Quality of life

#### Mutual and Shared Experiences

Mutual and shared experiences’ posts centered on participants sharing details regarding their own disabilities and personal stories. This was often as a way to inspire, motivate, and relate to others. The following is an example of 1 of these posts by a Facebook group member:

Just wonder of those disabled out there, are there others like me that only a few select family members support you? I have a spouse that has no compassion for me. Expects me to do everything, feels I am lazy, not in any pain! He resents the fact I receive benefits and don’t work. I’d give just about anything if I wasn’t ill and had no pain. I am looking for friends that know how I feel.

User post

People often responded with comments about how they are experiencing or have overcome a similar situation. Although not as frequent, some members posted on the Facebook group asking for support through sharing GoFundMe pages or similar financial support pages.

#### Societal Concerns

Posts classified under societal concerns included participants expressing their concerns regarding society’s interactions with their disability. Group members posted about concerns or excitement they had regarding inclusivity and accessibility in society:

I agree with you 100%. It’s the barriers we encounter through life. I never had trouble with kids as a child. But was segregated from things due to lack of access. Today it’s better as far as schools and public buildings. It has a long way to go. I encounter many places that I STILL cannot get in the bathroom or even the building. The American Disability Act exists here for that. But it is not enforced.

User post

#### Awareness

Awareness posts focused on events, helpful tools, and current research that could increase participants’ awareness about happenings in the disability community. The Facebook group allowed for the dissemination of content to raise awareness about several topics. For example, information about various types of disabilities was shared to inform people of technology they might not have heard about otherwise, often in the form of Web-based articles or news articles. Some of the posts and articles about technology included *No tie shoelaces for people with Autism or special needs*, *First paralyzed human treated with stem cells has now regained upper body movement*, and *The benefits of online therapy if you have a disability*. Relevant information about current research studies, prosthetics, and articles and videos about new types of treatments for persons with disabilities was also shared to increase awareness and knowledge of group members.

#### Health Care Policy

Health care policy posts in the Facebook support group voiced concerns regarding the impact of health care policy changes for persons with disabilities and current policy implementation in the United States. Many posts discussed implications for proposed policy changes to health insurance and other health care–related policies. In response to their concerns, members of the support group offered information and resources about Medicare and Medicaid to members in need. For example, 1 user posted a short explanation about how to lower the amount you are paying for Medicare:

To date, between 300 and 500 folks who were members and/or recently joined the Disability Digest have requested help with their Medicare Plans. It is estimated that their savings will be between $144,000 and $244,000 over the next year because they took advantage of our free health care consultation, with our health care experts. Now isn’t that a nice piece of change. The average savings per person is $40.00 a month, simply by getting into the correct Medicare Plan.

User post

Other common concerns that were shared and posted by group members involved government funding cuts and the current political administration.

### Online Discussion Forums

All posts from the online discussion forums were deidentified, and no user names were included in this paper. Quotes are presented as blockquotes with the participant identifier as [User post] after the quoted text to indicate an original post by a forum user. The same steps taken for the modification of Facebook posts were also taken for the modification of discussion forum posts. From the 116 discussion forums posts analyzed, 3 major themes were revealed: emotional outlet and support, health, and quality of life (Textbox 1).

#### Emotional Outlet and Support

The theme of emotional outlet and support was characterized by participants using connections provided through the forums for social support, advice seeking, and expressing emotions. Forum members posted about psychological stress and emotions and often received feedback and advice from others. For example, 1 member used a discussion forum as a space to express their process of self-realization to other members:

People that have dealt with difficulties are thought to be more compassionate and have empathy for those difficulties. I'm finding myself that in many ways this isn't really true. I'm learning that I can share my experiences and listen. It's not my job to fix their problems.

User post

This theme was also characterized by participants seeking everyday support through small talk with other forum members:

One thing I've found about [this forum] is that I can have a disagreement with another member and yet still remain friends. We can still give support to each other joke around together. This a very special place I'm thankful for it.

User post

#### Health

The theme of health can be described as participants utilizing the online discussion forum to share any physical, mental, or health care–related stories. It was common for forum users to post about their specific disability and the symptoms they experience. Many users were shown discussing health by describing their medical interactions. Medical interactions ranged anywhere from their disability diagnosis by a professional to symptom management. In the forums, participants also shared their personal medical diagnosis stories. For example, 1 user described their injury and disability sustained from a drunk driving incident:

I have four TBI [Traumatic brain injury] PTSD, herniated disks throughout my back and most of my neck. Alone with a variety of knee & hip injury. The joys of being hit by a drunk driver.

User post

In addition, many users mentioned mental health and mental illness in their posts. These posts comprised mental diagnosis disclosure, the emotions associated with such diagnoses, and seeking support from others with similar mental health experiences:

Well I have a learning disability (idk which one) along with depression, self-harm, and suicidal thoughts, and I been to a mental hospital twice…so that’s my junk I got to deal with…may I ask what disabilities you guys have? I’d like to know if anyone on this site is going through similar things. you don’t have to answer of course.

User post

#### Quality of Life

Discussion forum members posted on discussion boards about their perceived quality of life and how their disabilities affected their day-to-day lives in both positive and negative ways. Quality of life was shown to be influenced by perceptions about discrimination, accessibility and technology, family and relationships, and participation. One of the most common areas discussed was on discrimination. Participants posted about how acts of discrimination (ie, harassment or injustices done to persons with disabilities) and discrimination in community spaces (ie, neighborhoods, churches, and governmental agencies) could negatively impact an individual’s quality of life:

One day a female carrier and myself was in a discussion about school. She asked me about my income. I told her I don’t talk about with people because it's no one's business. She said it was her business because she works for the USPS. I told her in very unclean language how I see that, and left. She started mishandling my mail, so I filed for grievance.

User post

Forum members also shared about how accessibility could be positive or negative. Structural and environmental changes to make places more accessible for persons with disabilities were perceived as positive, but the lack of accessibility in most places was perceived as negative:

I am lucky enough to have moved in when the big adaptations were in place. I am extremely grateful for a fabulous wet room that I can access even on a wheelchair. The only addition made to this after I moved in was a “BIO BIDET”, this is a godsend for me with the personal problems I have, the simple act of being able to attend to your own toilet needs is a great boost to one’s self esteem.

User post

In addition, discussions forums were used as a way to share information with persons with disabilities about opportunities to participate in various activities and hobbies, such as jobs, sports, or entertainment, illustrating a willingness to help one another.

### Online Chat Rooms

The same steps taken for the modification of Facebook posts were also taken for the modification of chat room posts. In addition, for chat rooms specifically, it was common for multiple conversations to be going on at once. To eliminate confusion, the researchers deleted any comments not relevant to the ongoing conversations. Quotes from the online chat room data presented in this section are accompanied by anonymized identifiers. These were created to protect the identities of users.

From the 60 hours of data collected from the 2 online chat rooms, 3 major themes emerged: chat room interactions for emotional outlet and support, health, and quality of life (Textbox 1).

#### Emotional Outlet and Support

The theme of emotional outlet and support was characterized by chat room conversations where participants sought social support and interactions for physical, mental, and environmental struggles from other participants. The chat rooms served as spaces for participants to vent to one another, share feelings of distress and coping mechanisms, and receive feedback and advice from others when solicited. Moreover, they offered spaces for support through small talk and member interactions. Participants engaged in exchanges with other members by sharing information regarding everyday life, such as this interaction in one of the chat rooms seeking experiential advice about finding a job as a persons with disabilities:

Did you have a bad experience trying to find a job?

User A

I was being thrown many curved balls

User B

That with determination

User B

Sometimes people take my kindness as a weakness and they get surprised

User A

It can be done

User B

Some people do use people’s kindness to gain from

User B

Especially the people around my neck of the woods, give them an inch and they take a lightyear

User C

Yeah I admit I lost a lot of my confidence when I became disabled, but just running this household I am getting it back

User A

#### Health

The theme of health in online chat rooms was characterized by participants posting their daily physical, medical, and mental signs and symptoms of disabilities as a way of sharing their health experience with other users. Participants engaged in the online chat rooms by describing their specific disability, the symptoms associated with it, the way they managed their symptoms, and their interactions with medical professionals. The discussion below demonstrates the back-and-forth between users about their disability stories:

Are you disabled User E?

User D

Yup, multiple spinal diseases

User E

Sorry to hear

User D

I have crushed spinal cord

User D

No problem had quite a while to get used to it, its degenerative and very painful

User E

Try and stay happy lol

User E

You will get comfortable here…I have degenerative bone disease as well, in feet and moving up

User F

Accentuate the positive…

User G

Mine was lower back to start with affecting my legs & feet but now it’s in my neck causing problems with my arms & hands

User E

Mental health was another popular discussion topic among persons utilizing chat rooms, especially participants’ personal experiences with diagnosis, how their illness affected them, and coping mechanisms they use. This excerpt from a chat room exemplifies how users discussed mental health with one another:

I will admit I planned my funeral

User B

Some things that went through my head omg

User B

Still gets scary when legs you count on don’t respond to input

User D

Then you try hard to find comfort in routine and become dependent on what you expect to go smoothly as every other day but get hit with a sudden jolt and it wrecks your nerves and throws off your balance

User C

I call them curved balls

User B

But I think, why was that thrown at me

User B

Was having a good month so far then WHAM my bank account got hijacked

User C

#### Quality of Life

Quality of life chat room discussions centered around the individual’s perception on how disabilities positively or negatively affected their day-to-day life. Factors contributing to quality of life included jobs, finances, medical coverage, social support (eg, family and relationships), daily struggles, and issues regarding environment and accessibility. For instance, 1 chat room conversation was centered around an individual’s struggles with Medicare, prescriptions, and lack of information:

My Medicare keeps getting hacked for prescriptions, I have no idea how they get it but I have had several scripts filled in my name in Charlotte for different types of pain medication and Adderall

User A

It’s odd they are hitting small amounts

User C

They do that

User B

Damn that’s horrible

User C

Hoping you don’t notice

User B

Scammers everywhere, but I still see the good in mankind

User B

Well no one is doing anything about it, you need valid ID in NC to pick them up, but maybe they are fake, they tell me nothing. I just have to deal with it every time my doctor checks my records so he can fill mine

User A

Relationships were another aspect of quality of life that users were willing to disclose and discuss, often seeking counsel or solace:

Hey all, needing some help processing something at the moment. Not sure if this is the best place for this but here it goes. I found out today that my husband has been cheating. Any married gays out there with words of support?

User H

Chop his *explicative* off

User I

I don't really know what there is to say. But sitting thinking about it hasn't been productive for me

User H

Collect proof first, without letting on that you know

User K

Especially among participants in the chat rooms, personal information regarding family, relationships, and significant others was shared. Chat room members expressed how certain factors relating to family, relationships, and significant others influenced their quality of life.

## Discussion

### Principal Findings

In this study, we explored the ways in which online communities were utilized by persons with disabilities to facilitate communication interchange, disseminate information, and foster community support. The results indicate that persons with disabilities are utilizing the 3 platforms for various interactions (Table 2 and Textbox 2). On the basis of the findings of this study, the medium with which the individual is interacting (eg, Facebook, discussion forums, or chat rooms) influences the individual’s interactions. They are likely intentionally choosing to interact on a platform based on the functions it offers. It is possible that specific platforms serve specific purposes that may not be interchangeable [25].

**Table 2:** Differences between how persons with disabilities used the 3 social networking sites.

Differences across platforms	Social networking site		
	Facebook	Discussion forums	Chat rooms
Type of social support	Informational, esteem and emotional, and instrumental	Informational, esteem and emotional, and belonging	Informational, esteem and emotional, and belonging
Format	Structured	Less structured and informal	Less structured and informal
Topics of discussion	News stories, raising awareness, and advocating for persons with disabilities; very little mention of mental health	Advice seeking, emotional processing and stress relief, and day-to-day experience of living with a disability; almost daily discussions of mental health	Small talk, emotional processing and stress relief, disability disclosure, and day-to-day experience of living with a disability; almost daily discussions of mental health
Familiarity with group members	Little sense of familiarity among members	Some sense of familiarity among members	Greatest sense of familiarity among members
Depth of content	Surface level sharing	Surface level to medium-depth sharing	In-depth sharing
Type of interactions	Mostly positive responses	Mostly positive responses with some negative responses	Positive and negative responses
Personal identifiers	Information shared and discussed was linked to personal Facebook accounts	Anonymous	Anonymous

**Similarities between how persons with disabilities used the 3 social networking sites.**

Similarities across all platforms:

- Members both request and provide information
- Members sympathize with one another
- Support through shared experiences
- Platforms serve as safe spaces for sharing among members
- Functional social support present among each type of social networking site

The Facebook support group for persons with disabilities emphasized mutual and shared experiences, societal concerns, raising awareness, and concerns about health care policy in the United States. People in the Facebook group seemed willing to be somewhat vulnerable within this online community setting, sharing stories of personal distress, independence, and support (or lack thereof); however, interactions within the Facebook group appeared much more structured and superficial than discussion forums or chat rooms (Table 2). The perceived injunctive norms might contribute to the structure and superficiality among Facebook support groups [30,31]. In addition, the Facebook group provided a safe space for users to respond to news stories and articles about abuse, violence, and discrimination against persons with disabilities as well as share their concerns or excitement regarding inclusivity and accessibility in society (Table 2 and Textbox 2). It is possible that this platform allows people to share about certain topics without fear of criticism, negativity, or backlash from others. This may not be the case if one of them were to post similar content on their personal Facebook timeline.

The most common forms of interaction between members of the Facebook group included requesting and providing information, sympathizing with other users, raising awareness and advocating for persons with disabilities, and generating support through shared experiences and concerns (Table 2). The interactions between members correspond to both informational and esteem and emotional social support (Table 2). These findings align with research conducted by Mustafa et al [24] who found that Facebook support groups for parents with Autism Spectrum Disorder were commonly used for informational support, emotional support, and sharing of personal experiences. The findings are also in line with a different study, which found that Facebook, as an SNS, was an online environment well suited for the exchange of informational support [20]. Although not as common, instrumental support was sought by some Facebook group members. This was exemplified by members sharing their financial support pages, most likely as a request to receive instrumental support from the online community (Table 2).

In comparison, the interactions within online discussion forums and chat rooms were less structured than the Facebook support group and more similar in the way persons with disabilities utilized them (Table 2). The same 3 themes emerged in both the platforms: emotional outlet and support, health, and quality of life. Both platforms were most commonly used as venues for persons with disabilities to relieve psychological stress, express themselves emotionally, and share and vent about their experiences in hopes of receiving positive support, feedback, and relief from others (Table 2). Members seemed especially grateful for the existence of a safe space where mundane, everyday interactions could take place, and yet, the individuals still felt a sense of belonging and support by their community (Table 2 and Textbox 2). Although both platforms exhibited interactions characterized by members responding both positively and negatively to others, these types of interactions were most common among online chat room users (Table 2). These interactions seemed accepted, and expected, by members of the chat room, exemplifying a comfort among chat room users to offer support in the most candid ways possible. This familiarity seemed to encourage real and honest connections between users, similar to a group of tight-knit friends (Table 2). These findings demonstrate the existence of esteem and emotional, belonging, and informational social support among discussion forums and chat rooms (Table 2).

Interactions in discussion forum and chat room settings are anonymous, a feature that has been found to be valuable to persons with disabilities on these platforms [14,23,25,32] (Table 2). Anonymity is not a feature of Facebook, as your profile is linked to your name and usually includes a picture of yourself (Table 2). It is possible that interactions are more candid and unstructured among forums and chat rooms because of the comfort and protection anonymity can provide for users [14,16,25,32]. This could also explain the prevalence of discussions surrounding mental health issues on both forums and chat rooms and the lack of such discussion on the Facebook group (Table 2). Members of the forums and chat rooms mentioned their experiences with mental health almost daily, whereas members of the Facebook group seldom posted about mental health and mental illness (Table 2). Mental health and mental illness discussions were most prevalent among chat room users compared with any of the other support groups.

In general, chat room discussions of mental health and mental illness were more in-depth and descriptive, with participants offering more personal details (Table 2). Participants logged into the chat room would respond to others in supportive, consoling ways and attempt to offer helpful advice to disclosing users. Discussion forum posts were more surface-level in their discussion of mental health and mental illness, with participants offering little insight into their own personal experiences (Table 2). Facebook group posts were mostly aimed at spreading awareness as opposed to disclosing one’s own personal experience with mental illness (Table 2). These findings suggest that a safe and anonymous online environment might help facilitate engagement in discussions about commonly stigmatizing and taboo topics.

### Limitations

This study is not without limitations. First, the data are subjective based on the researchers’ interpretations, as is the nature of qualitative research. The importance of using qualitative methods for this study, however, should not be understated. The content gained from a qualitative process provides a more in-depth and nuanced understanding of how Facebook support groups, discussion forums, and chat rooms are used by persons with disabilities. Although qualitative studies are relevant and important in their own right, quantitative research is also necessary as a next step. Future research could investigate how the effectiveness of online social support can be maximized for persons with disabilities. Second, as only 1 Facebook online support group was analyzed in this study, we cannot generalize these findings to all support groups on Facebook. Discussion within this Facebook group was not necessarily encouraged between members, which might indicate a more passive approach to participation among Facebook support group users. This could potentially limit the usefulness of support offered through this domain or influence the type of support that is perceived or received. It is possible that users seek out support groups on Facebook because of this type of participation; however, further research is required to understand how and why users seek out certain forms of online support and the roles active and passive participation play in the overall perception and reception of social support. Third, because of the predetermined date constraints and the difficulty collecting data from live chat rooms, only a few groups for each type of SNS were analyzed and compared. As this was a pilot study, the number of groups per SNS needed for the researchers to capture a snapshot of what was occurring in online disability support groups was small. In the future, more groups should be analyzed and compared to enhance the explanatory power and generalizability of the study. It might also be important to investigate online social support among SNS groups as it relates to specific disabilities. Future studies could compare online support groups among these 3 SNS by type of disability to understand the similarities and differences of how individuals with varying disabilities interact. This could provide an even more nuanced understanding into the ways SNS are used for social support by persons with disabilities.

### Implications of Findings

This study allows us to focus on and determine beneficial ways to incorporate SNS into treatment, foster social support, and develop awareness among the community of persons with disabilities. Future directions highlight the potential to intervene with this population through social network mediums. Future interventions can be developed utilizing Facebook, discussion forums, and chat rooms as mediums, depending on the desires and needs of the population of persons with disabilities. Moreover, the reach of these Web-based interventions and groups can be easily assessed because of increasing ease of access to the internet.

Online support groups are not bounded by space constraints. They are a medium where perspectives, experiences, and viewpoints are welcomed, and diversity is encouraged, while also promoting a feeling of universality among members [32]. Online support groups offer the ability to increase support for persons with disabilities while simultaneously breaking down the geographic, transportation, and stigmatizing barriers this population faces. Facebook support groups, discussion forums, and chat rooms represent 3 unique platforms where social support can be facilitated via social networks. Interactions on each of the platforms displayed elements of each of the 4 types of social support (esteem and emotional, informational, instrumental, and belonging; Textbox 2) [6,9], indicating the ability for social support to be facilitated among SNS.

As social support is being provided (in varying ways) on each of the online platforms studied (Table 2 and Textbox 2), it is important to understand how online social support might influence the health and well-being of persons with disabilities. Online social support has been suggested to have similar benefits to those of in-person social support, including benefits to health and well-being [6,16,18,25,33]. Disability research has shown that social support has the ability to either alleviate or exaggerate disability symptoms depending on several factors [34]. According to the 3 theoretical models of social support [9,10], social support can influence health in many ways. As the nature of the study was qualitative, we could only speculate how the interactions of persons with disabilities via these platforms might have positively influenced their health.

The stress prevention model posits that social support may provide an individual with resources to avoid or reduce exposure to certain stressors. Reduced exposure to stressors, in turn, is associated with enhanced health [9,10]. Through the facilitation of social support among online communities, group members may be able to provide other persons with disabilities with the resources they need to avoid or reduce their exposure to some types of stressors. This could be by influencing cognitive processes, encouraging proactive coping, or decreasing exposure to secondary stressors [9]. There is also the stress buffering model, which proposes that social support can act as a stress-buffering agent. In this model, social support provides resources that help an individual appropriately cope with stress, which buffers the association between stress and health-related outcomes [9,10,15]. The provision of social support from online support group members could enhance one’s coping resources and interpretation of their stressful situation, thus weakening the harmful effects of stress on health and well-being [9,15]. Finally, the direct effect model suggests that social support is effective in a more general sense, regardless of stress. This model is concerned with the ways in which membership in a social network and an individual’s sense of connection has an overall beneficial effect on their well-being, health, and health-related outcomes [9,10,15]. By being a member of an online support group, persons with disabilities might have, overall, a greater sense of connection and feel cared for and supported by others [9].

SNS allow persons with disabilities to mobilize social support in ways similar to traditional offline settings and in ways that are unique to online contexts. As social platforms continue to develop, grow, and evolve, they have the potential to help reduce, and possibly eliminate, many of the barriers to social support experienced by persons with disabilities. In doing so, these platforms could have lasting impacts on both their health and well-being.

## Abbreviations

SNS: social networking sites
